# Optimism, Positive and Negative Affect, and Goal Adjustment Strategies: Their Relationship to Activity Patterns in Patients with Chronic Musculoskeletal Pain

**DOI:** 10.1155/2018/6291719

**Published:** 2018-03-15

**Authors:** Rosa Esteve, Alicia E. López-Martínez, Madelon L. Peters, Elena R. Serrano-Ibáñez, Gema T. Ruiz-Párraga, Carmen Ramírez-Maestre

**Affiliations:** ^1^Andalucía Tech, Facultad de Psicología, Instituto de Investigación Biomédica de Málaga (IBIMA), Universidad de Málaga, Málaga, Spain; ^2^Department of Clinical Psychological Science, Faculty of Psychology and Neuroscience, Maastricht University, 616 6200 MD Maastricht, Netherlands

## Abstract

**Objective:**

Activity patterns are the product of pain and of the self-regulation of current goals in the context of pain. The aim of this study was to investigate the association between goal management strategies and activity patterns while taking into account the role of optimism/pessimism and positive/negative affect.

**Methods:**

Two hundred and thirty-seven patients with chronic musculoskeletal pain filled out questionnaires on optimism, positive and negative affect, pain intensity, and the activity patterns they employed in dealing with their pain. Questionnaires were also administered to assess their general goal management strategies: goal persistence, flexible goal adjustment, and disengagement and reengagement with goals.

**Results:**

Structural equation modelling showed that higher levels of optimism were related to persistence, flexible goal management, and commitment to new goals. These strategies were associated with higher positive affect, persistence in finishing tasks despite pain, and infrequent avoidance behaviour in the presence or anticipation of pain.

**Conclusions:**

The strategies used by the patients with chronic musculoskeletal pain to manage their life goals are related to their activity patterns.

## 1. Introduction

Patients make substantial adaptive efforts to deal with chronic pain. In their continuous attempts to manage chronic pain, they usually change the way in which they engage in daily activities. However, the goal of pain management is just one of the goals to be pursued in a context of other concomitant goals [1–5]. The specific strategies that patients use to manage these different and sometimes opposing goals may determine their behaviour when dealing with daily activities (i.e., the so-called activity patterns).

### 1.1. Activity Patterns in Patients with Chronic Pain

Traditionally, three activity patterns have been distinguished: avoidance, persistence, and pacing. However, more specific activity patterns have been identified in patients with chronic pain [6, 7]. Avoidance has been divided into two patterns: (a) pain avoidance, which refers to avoidance behaviour in the presence or anticipation of changes in pain (e.g., “I stop what I am doing when my pain starts to get worse”), and (b) activity avoidance, which refers to the patients' condition of being in pain rather than the fluctuating pain experience (e.g., “I have not been able to carry on with my usual level of activity”). Research has shown that activity avoidance is associated with poorer physical and psychological functioning, whereas pain avoidance is not related to patient adjustment [6, 7]. Three types of persistence have been differentiated: (a) task-contingent persistence, in which patients persist in finishing tasks or activities despite pain (e.g., “Once I start an activity I keep going until it is done”); (b) excessive persistence, referring to doing too much, not respecting one's physical limits (e.g., “I find myself rushing to get everything done before I crash”); and (c) pain-contingent persistence, in which the level of activity fluctuates with and is determined by the pain at that moment (e.g., “When my pain decreases I try to be as active as possible”). A recent study of a sample of patients with musculoskeletal pain found that all three types of persistence were positively associated with daily functioning [7]. Finally, pacing is characterized by dividing daily activities into smaller tasks. Three types of pacing have been distinguished according to the goal of the behaviour: (a) to increase activity levels (e.g., “I usually take several breaks and so I can do a lot more things”), (b) to conserve energy for valued activities (e.g., “I split activities into smaller steps so I can save energy to do other things that matter to me”), and (c) to reduce pain (e.g., “I split activities into smaller steps so that it hurts less”).

### 1.2. Activity Patterns from a Motivational Perspective

It has been emphasized that a theoretical model is needed to explain why patients with chronic pain engage in different activity patterns (i.e., the motivational mechanisms underlying activity patterns) [8]. Chronic pain interferes with daily activities and goals, and consequently, patients may need to negotiate the competition between their goals for limited physical and cognitive resources. From this point of view, activity patterns are viewed not solely as a product of pain but also as the result of the self-regulation of current goals in the context of pain [8]. It is therefore relevant to study the relationship between the strategies that patients with chronic pain use to manage their goals and their activity patterns.

### 1.3. Goal Management Strategies

Three goal management models can be distinguished. Firstly, the dual process model [9] differentiates two complementary strategies: the *assimilative mode* (tenacious goal pursuit), which is directed at maintaining goals by intentional efforts that modify the actual situation in accordance with personal goals, and the *accommodative mode* (flexible goal adjustment), which is directed at adjusting goals to situational or physical constraints. Several studies in patients with a range of chronic conditions, including chronic pain, have found that the combined use of accommodative and assimilative strategies was associated with well-being [10–12].

Secondly, the goal adjustment theory [13, 14] describes possible reaction patterns when goals are no longer attainable. This theory proposes that adjustment entails both *disengaging* from unattainable goals and *reengaging* in alternative goals. This theory has been strongly supported by empirical research, showing that individual differences in the capacity to adjust to unattainable goals predict both subjective well-being and physical health.

Thirdly, the two aforementioned theories have recently been combined in the integrated model of goal management [15]. According to this model, the adaptive value of a given goal management strategy depends on the patients' situation. Goal maintenance is the preferred strategy when a person still perceives opportunities to attain a goal. Goal adjustment—understanding goal disengagement as a form of goal adjustment—is more suitable for situations in which goals are under threat. Goal reengagement appears to be an appropriate strategy at all times and can complement existing goals or replace unattainable goals. This model has been tested in a sample of patients with arthritis [15], finding that patients who reported a lower tendency to adjust their goals had higher anxiety and depression scores. Patients who reported a greater tendency to adjust their goals to changed circumstances experienced more purpose in life, more positive affect, and were more satisfied with their participation in daily life activities.

### 1.4. Optimism as a Facilitator of Goal Adjustment

Optimism reflects the extent to which people hold generalized favourable expectations for their future [16]. In relation to pain, recent clinical and experimental evidence suggests that positive affect and optimism are two of the most important resilient resources for successful adaptation to acute and chronic pain [17–27]. Although there is considerable evidence linking optimism and favourable outcomes, additional research is needed to better understand the mechanisms underlying the effect of optimism on health and well-being [28]. In this line, several studies have shown that optimism could promote well-being through the facilitation of goal adjustment [29]. Optimists are more inclined than pessimists to pursue goals tenaciously [30], although they also engage in flexible goal adjustment [31]. Moreover, they are more likely to reengage in new goals when their current goals are not attainable [29, 32].

### 1.5. Affect and Goal Adjustment

A factor that could facilitate disengagement from unattainable goals and reengagement with new goals is the perception of available alternatives, which could be promoted by positive affect. In contrast, negative affect could narrow the perception of available alternatives and consequently could be related to disengagement from unattainable goals. As the “broaden-and-build theory” postulates, positive affect broadens attention to other stimuli, thoughts, and opportunities and facilitates the ability to think creatively and flexibly [33]. This may explain the finding that, in contrast to negative affect, positive affect is related to a more diverse array of goal management strategies [12, 29, 31, 32, 34, 35]. Thus, the “broaden-and-build theory” could complement goal regulation models: given that optimism and pessimism are related to positive and negative affect, respectively, it could be postulated that affect mediates the relationship between optimism and pessimism and goal management strategies. However, affect may not only be an antecedent of goal management strategies but may also result from the specific strategy employed [10–15] and/or from the ensuing activity patterns [6, 7]. Thus, it seems relevant to study the specific role that affect may play in the relationship between optimism/pessimism, goal management strategies, and activity patterns.

Briefly, longitudinal evidence shows that disengagement is negatively related to negative affect because successful disengagement could contribute to the quality of life by avoiding the stress of repeated failures [36]. On the other hand, positive affect is positively associated with tenacious goal pursuit, flexible goal adjustment, and goal reengagement [10–15].

### 1.6. Activity Patterns and Affect

Previous research has shown that positive affect is positively associated with nonpain-centred activity patterns (task-contingent persistence, pacing to increase activity levels, and pacing to conserve energy for valued activities), whereas negative affect is positively related to pain-centred activity patterns (activity avoidance, pain avoidance, pain-contingent persistence, and pacing to reduce pain) [6, 7].

### 1.7. Aims and Hypotheses

**Figure 1:**
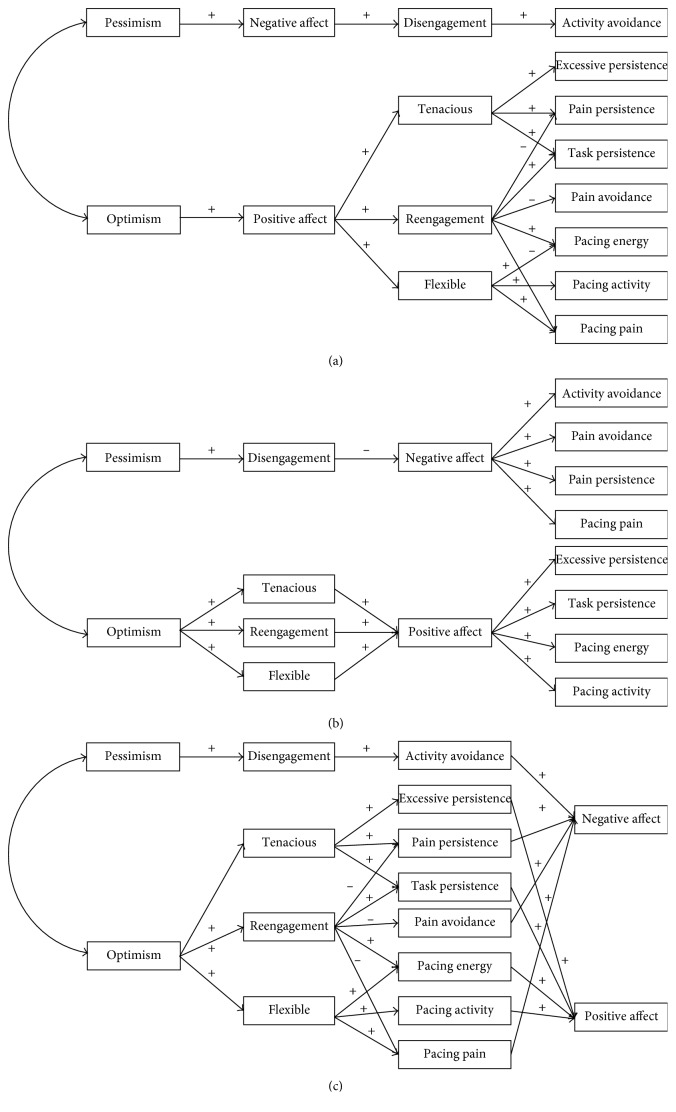
Hypothetical alternative models. (a) Model 1, (b) Model 2, and (c) Model 3.

The aim of the present study was to investigate the association between goal management strategies and activity patterns in patients with chronic musculoskeletal pain while taking into account the role of optimism/pessimism and positive/negative affect. Three alternative models were tested. Firstly, it was postulated that affect would mediate optimism/pessimism and goal management strategies which, in turn, were hypothesized to be associated with activity patterns (Model 1). Secondly, it was postulated that there would be a direct relationship between optimism/pessimism and goal management strategies, and that affect would mediate goal management strategies and activity patterns (Model 2). Finally, it was postulated that optimism/pessimism would be related to goal management strategies, and that goal management strategies would be associated with activity patterns which, in turn, would be related to affect (Model 3).  Figure 1 shows the three models. A detailed description of the postulated relationships is included in Data Analysis.

In the field of chronic pain, research based on goal management models is scarce. As far as we know, this study is the first to investigate the relationship between optimism, pessimism, positive and negative affect, goal management strategies, and activity patterns in patients with chronic musculoskeletal pain.

## 2. Methods

### 2.1. Procedure

This study formed part of a larger research project [7, 37], which was approved by the University of Málaga Ethics Committee. Participants were recruited through a physiotherapy unit and two local associations of patients with fibromyalgia and by doctors working at the Hospital Costa del Sol Pain Unit and the Hospital Quirón Rheumatology Unit in Málaga. The data were collected between March 2016 and December 2016. Individuals were considered eligible for inclusion if they met the following criteria: at the moment of participation in the study, they were experiencing musculoskeletal pain and had been experiencing pain for at least the last 6 months; they were between 18 and 65 years; they were not being treated for a malignancy, terminal illness, or psychiatric disorder; they were able to understand the Spanish language (spoken and written); and they were able to understand the instructions and questionnaires. The patients were informed of the study aims, confidentiality was assured, and written informed consent was obtained. Each participant had a semistructured interview with a psychologist to obtain demographic, social, and medical history data. Subsequently, they completed self-report questionnaires in the order described in Variables and Instruments.

Two psychologists took part in data collection. They were trained in the application of the protocol to guarantee the standardization of the assessment process and were blinded to the study design and hypotheses. The patients were always assessed in their usual health centre or in the facilities of the associations. Each session lasted approximately 1 hour.

### 2.2. Participants

Three hundred and eighty-eight patients were invited to take part in the study. Of these patients, 98 refused participation, 32 did not meet the inclusion criteria, and 21 were eliminated after preliminary analyses because they were outliers.

The final sample comprised 237 chronic musculoskeletal pain patients (192 women and 45 men). The average age was 52 years (SD = 9.95). At the time of the study, 71.30% were married or cohabiting. Regarding employment, 39.80% were active workers, 23.30% were retired, 20.30% were unemployed, and 15.3% were homemakers.

A total of 31.90% had completed high-school education and 39.60% had completed primary education. The median pain duration was 12.16 years (SD = 18.88), and the average pain intensity was 6.54 (SD = 1.33). The participants had musculoskeletal pain at different locations: generalized pain conditions were the most frequent (44.52%) (fibromyalgia, 28.27%; generalized osteoarthritis, 12.72%; and other conditions, 3.52%), followed by spinal pain, 26.14% (cervical, 3.53%; lower back, 6.01%; and other back sites, 16.61%), pain in the upper shoulder and upper limbs (15.19%), and pain in the lower limbs (14.13%).

### 2.3. Variables and Instruments

#### 2.3.1. Dispositional Optimism

Dispositional optimism was assessed using the Spanish version of the Life Orientation Test-Revised (LOT-R) [38, 39]. The LOT-R consists of six scored items (items 1, 4, and 10 are positively worded and items 3, 7, and 9 are negatively worded) plus four filler items. The optimism and pessimism subscale scores were calculated by summing the positive and negative items, respectively. Respondents indicate the extent to which they agree with each item on a 5-point Likert-type scale ranging from 0 (strongly disagree) to 4 (strongly agree). In the present study, the LOT-R total score had Cronbach's alpha of 0.90. Cronbach's alpha for the optimism and pessimism subscales was 0.85 and 0.81, respectively. The Spanish LOT-R has shown adequate criterion validity [40].

#### 2.3.2. Positive and Negative Affect

Positive and negative affect was assessed using the Spanish version of the Positive and Negative Affect Schedule (PANAS) [41–43], which is one of the most reliable, valid, and efficient means to measure these variables. It comprises two 10-item scales. The instrument has demonstrated appropriate stability over a 2-month time period. The Spanish PANAS also has excellent construct and criterion validity. In this study, the positive affect and negative affect scales had Cronbach's alpha of 0.90 and 0.87, respectively.

#### 2.3.3. Pain Intensity

Patients were asked to rate their mildest, average, and worst pain during the past 2 weeks, as well as their current pain, on a scale ranging from 0 to 10, with “0” indicating “no pain” and “10” indicating “pain as intense as you could imagine.” A composite pain intensity score was calculated for each participant by calculating the average of the mildest, average, worst, and current pain [44].

#### 2.3.4. Activity Patterns

The Activity Patterns Scale [7] consists of 24 items grouped into 8 three-item subscales: pain avoidance (*α* = 0.72), activity avoidance (*α* = 0.82), task-contingent persistence (*α* = 0.87), excessive persistence (*α* = 0.80), pain-contingent persistence (*α* = 0.92), pacing to increase activity levels (*α* = 0.72), pacing to conserve energy for valued activities (*α* = 0.81), and pacing to reduce pain (*α* = 0.78). The participants are asked to indicate to what extent the statement applies to them on a 5-point scale ranging from 0 (not at all) to 4 (always). The instrument showed adequate reliability as well as structural, convergent, and criterion validity [7].

#### 2.3.5. Goal Management Strategies

The Goal Disengagement and Goal Reengagement Scale [14] is a 10-item instrument that measures the individual's usual reaction to having to stop pursuing an important goal. The instrument comprises a 5-point Likert-type scale ranging between 1 (almost never true) and 5 (almost always true). Four items measure an individual's tendency to disengage from unattainable goals (e.g., “It's easy for me to reduce my effort toward the goal,” or “I stay committed to the goal for a long time; I can't let it go”) and six items measure an individual's tendency to reengage with new goals (e.g., “I seek other meaningful goals,” or “I start working on other new goals”). The Spanish version of the instrument has adequate criterion validity, internal consistency, and stability, and its factor structure is similar to the original structure [45]. In this study, the Goal Disengagement and the Goal Reengagement Scales had Cronbach's alpha of 0.70 and 0.94, respectively.

The Tenacious Goal Pursuit and Flexible Goal Adjustment Scales [9] assess two distinct modes of coping with goal disruption, respectively: tenacious goal pursuit (e.g., “The harder a goal is to achieve, the more desirable it often appears to me”) and flexible goal adjustment (e.g., “In general I do not stay upset for long when I miss an opportunity”). Respondents rate the degree to which they agree with each statement on a 5-point Likert scale ranging from “fully disagree” to “fully agree.” The exploratory factor analysis of the Spanish version of the scales showed the same number of factors as the original scales and was ratified by confirmatory factor analysis. Cronbach's alpha, test-retest reliability, and correlations between the scales were also similar to the original scales. The scales also demonstrated adequate criterion validity [45]. In this study, the Tenacious Goal Pursuit and the Flexible Goal Adjustment Scales had Cronbach's alpha of 0.80 and 0.81, respectively.

### 2.4. Data Analysis

Statistical analyses and structural equation modelling (SEM) were conducted using SPSS 15.0 software and LISREL 8.80 software, respectively [46]. Mean scores, standard deviations, and correlation coefficients for all variables were calculated.

The fit of each of the three hypothetical models (Figure 1) was tested using SEM. The data were checked prior to the analyses. Outliers were identified by cluster analysis-based outlier detection in which each record is assigned an anomaly index, which is the ratio of the group deviation index to its average over the cluster that the case belongs to [47]. Twenty-one participants were excluded from the sample because they presented anomalous values for one of the variables included in the model. We also found that some variables were not normally distributed; thus, we used maximum likelihood as the estimation method because this method is effective for any data distribution when the analyses are performed on covariance matrices, and the matrix of fourth-order moments is provided [48].

The goodness-of-fit indexes used for the overall model were Satorra-Bentler chi-square [49], the Comparative Fit Index (CFI) [49], the Normed Fit Index (NFI) [50], the root mean-square error of approximation (RMSEA), and the Akaike Information Criterion (AIC) [51]. Satorra-Bentler chi-square is a chi-square fit index that corrects the statistic under distributional violations. In order to reduce the sensitivity of chi-square to sample size, the index is divided by the degrees of freedom [49]. Ratios of 2 or smaller are indicative of an acceptable fit of the model [52]. The CFI and NFI measure the proportional improvement in fit by comparing a hypothesized model with the null model as the baseline model. The CFI and NFI range from 0 (absolute lack of fit) to 1 (perfect fit), and fit is considered to be good when the values are more than 0.90 [53]. The RMSEA is an absolute misfit index; the closer to zero, the better the fit. Values less than 0.08 indicate an adequate fit, and values less than 0.06 indicate a good fit [53, 54]. Finally, the AIC index allows alternative models to be compared by taking into account parsimony (in the sense of the number of parameters) as well as fit. This index can be used regardless of whether or not the models can be ordered in a nested sequence. In this approach, the models are ranked according to their AIC values, and the model with the smallest value is chosen [51].

Three alternative models were tested (Figure 1). Age and pain intensity were used as control variables in the three models. These variables were used as covariates in the models for the variables with which they were significantly correlated (age and goal reengagement, pain intensity and flexible goal adjustment, positive and negative affect, pain avoidance, activity avoidance, task-contingent persistence, and pacing to reduce pain). In the three models, optimism and pessimism are the exogenous variables (i.e., variables not determined by any other variable in the model; Figure 1). The remaining variables are endogenous (i.e., variables determined by one or more variables in the model). All residual variances were assumed to be uncorrelated, and the exogenous variables were assumed to be correlated.

Causal paths were defined according to the hypothetical structural equation models shown in Figure 1. Path coefficients should not be interpreted as correlation coefficients. For example, a path coefficient of 0.80 connecting two variables (A and B) means that if A increases by one standard deviation from its mean, B would be expected to increase its own standard deviation by 0.80, while all other relevant connections remain constant. A path coefficient of −0.16 means that if A increases by one standard deviation from its mean, B would be expected to decrease its own standard deviation by 0.16, while all other relevant connections remain constant. The following paths were postulated for each of the models tested:

*Model 1*: (a) higher pessimism and higher optimism would be associated with higher negative effect and higher positive affect, respectively; (b) higher negative affect would be associated with the more frequent use of the disengagement goal management strategy; (c) higher positive affect would be associated with the more frequent use of tenacious goal pursuit, flexible goal adjustment, and goal reengagement strategies; (d) goal disengagement, understood as a tendency to abandon unattainable goals, would be positively associated with the activity avoidance pattern in which individuals give up doing things due to pain; (e) goal reengagement, understood as a tendency to commit to new goals, would be inversely related to activity patterns in which the patients are only centred on the goal of pain management (i.e., pain avoidance, pain-contingent persistence, and pacing to reduce pain). In contrast, goal reengagement would be positively associated with task-contingent persistence and with pacing to conserve energy for valued activities because these patterns imply that individuals are committed to goals other than pain control; (f) tenacious goal pursuit would be positively associated with task-contingent persistence, pain-contingent persistence, and excessive persistence; and (g) flexible goal adjustment would be positively associated with the three types of pacing because pacing involves adapting behaviour to the situational constraints without giving up the final goal (e.g., by flexibly alternating between rest and activity).
*Model 2*: (a) higher pessimism would be associated with higher disengagement; (b) higher optimism would be associated with the more frequent use of tenacious goal pursuit, flexible goal adjustment, and goal reengagement strategies; (c) higher disengagement would be related to lower negative affect; (d) higher tenacious goal pursuit, flexible goal adjustment, and goal reengagement strategies would be associated with higher positive affect; (e) higher positive affect would be related to higher task-contingent persistence, higher excessive persistence, higher pacing to increase activity levels, and higher pacing to conserve energy for valued activities; and (f) higher negative affect would be associated with higher activity avoidance, higher pain avoidance, higher pain-contingent persistence, and higher pacing to reduce pain.
*Model 3*: (a) higher pessimism would be associated with higher disengagement; (b) higher optimism would be associated with the more frequent use of tenacious goal pursuit, flexible goal adjustment, and goal reengagement strategies; (c) goal disengagement would be positively associated with the activity avoidance pattern; (d) goal reengagement would be inversely related to pain avoidance, pain-contingent persistence, and pacing to reduce pain and would be positively associated with task-contingent persistence and pacing to conserve energy for valued activities; (e) tenacious goal pursuit would be positively associated with task-contingent persistence, pain-contingent persistence, and excessive persistence; (f) flexible goal adjustment would be positively associated with the three types of pacing; (g) higher task-contingent persistence, higher excessive persistence, higher pacing to increase activity levels, and higher pacing to conserve energy for valued activities would be related to higher positive affect; and (h) higher activity avoidance, higher pain avoidance, higher pain-contingent persistence, and higher pacing to reduce pain would be related to higher negative affect.

## 3. Results

Table 1 shows mean scores, standard deviations, and correlation coefficients for all measures.

Table 2 shows all the GFIs of the 3 models tested via SEM. As can be seen, the three models meet the recommended cutoff criteria. The AIC index showed that the Model 2 had the smallest value and thus the best fit.

**Figure 2:**
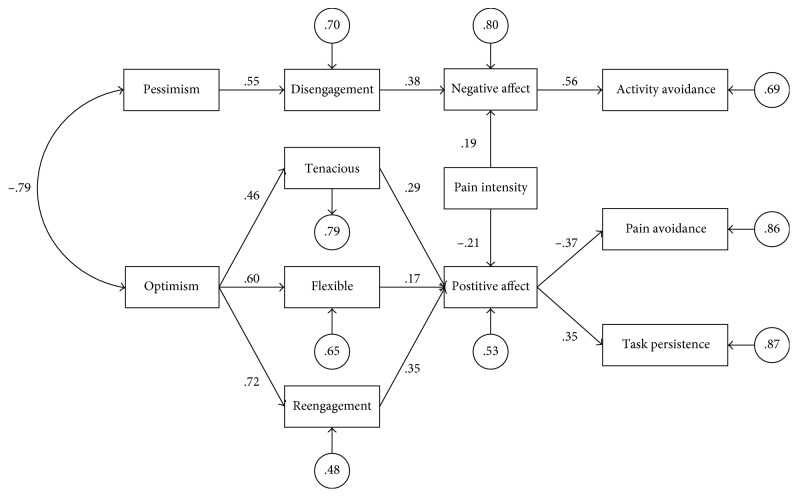
Final model. Rectangles represent observed (measured) variables, circles represent standardized error variances, straight lines with arrows represent presumed causal paths, values above the arrows represent standardized path coefficients, and the curved line represents the correlation between the exogenous variables.

Thus, Model 2 was taken as the starting point for further modification. Modifications were sequentially made in line with the recommendations of the Lagrange multiplier test [48]. Firstly, we deleted all paths of the initial model that were not statistically significant. For this reason, the variables excessive persistence, pain-contingent persistence, pacing to increase activity levels, pacing to conserve energy for valued activities, and pacing to reduce pain were excluded from the model. Except for pain intensity, in relation with positive and negative affect, the remaining covariates were excluded from the final model. Secondly, a relationship suggested by the modification indexes was included: a path from positive affect to pain avoidance.  Figure 2 shows the final model.

All path coefficients were statistically significant (*P* < 0.05). The goodness-of-fit indexes indicated that the estimated final model provided a good fit to the data. The Satorra–Bentler chi-square (10.67) divided by the degrees of freedom (50) was 0.21, which indicated an adequate fit of the model. The CFI (1) and NFI (1) had values higher than 0.90, which indicated an adequate fit. The RMSEA was 0.00 (values less than 0.06 indicate a good fit).

As shown in Figure 2, a high negative correlation was found between optimism and pessimism. Pessimism yielded a statistically significant positive path coefficient to disengagement, disengagement yielded a statistically significant positive path coefficient to negative affect, and negative affect yielded a significant positive path coefficient to activity avoidance. Optimism yielded three statistically significant positive path coefficients to tenacious goal pursuit, flexible goal adjustment, and goal reengagement. Tenacious goal pursuit, flexible goal adjustment, and goal reengagement each yielded a statistically significant positive path coefficient to positive affect. Positive affect yielded a statistically significant positive path coefficient to task-contingent persistence and a statistically significant negative path coefficient to pain avoidance. Finally, pain intensity yielded a statistically significant positive path coefficient to negative affect and a statistically significant negative path coefficient to positive affect.

## 4. Discussion

The aim of the present study was to investigate the association between goal management strategies and activity patterns in patients with chronic musculoskeletal pain while taking into account the role of optimism/pessimism and positive/negative affect. Special attention was paid to the role of positive/negative affect. Three alternative models were tested in which affect was hypothesized to play different roles.

The model with the best fit was the one in which a direct relationship was postulated between optimism/pessimism and goal management strategies and in which affect was hypothesized to mediate goal management strategies and activity patterns. Specifically, in the face of unattainable goals, patients with chronic pain and higher levels of pessimism reported that they tended to abandon such goals (disengagement). This strategy was associated with higher levels of negative affect which, in turn, was related to the more frequent use of the pattern of activity avoidance (i.e., the abandonment of activities because of the pain condition). On the other hand, patients with chronic pain characterized by higher levels of optimism reported being more persistent in pursuing their goals, more able to adjust their goals to situational constraints, and in the face of unattainable goals, to more easily commit to new goals (reengagement). The more frequent use of these three strategies was associated with higher positive affect which, in turn, was related to the more frequent use of a pattern of activity characterized by persistence in finishing tasks or activities despite pain. Furthermore, positive affect was related to the more infrequent use of a pattern characterized by avoidance behaviour in the presence or anticipation of changes in pain.

### 4.1. Optimism/Pessimism and Goal Management Strategies

The findings of this study are in line with those of previous research showing that optimists and pessimists cope in different ways to threats to their health [29]. According to the results, patients with chronic musculoskeletal pain who have positive expectations for their future exert continuing effort when the achievement of their goals is threatened. Nevertheless, it must be taken into account that excessive persistence in the face of failure could lead to resource depletion and frustration [55]. Successful adaptation requires combining tenacity with a certain amount of flexibility; for example, chronic musculoskeletal pain sometimes demands the reformulation of the patients' current goals or a change in the strategies used to achieve such goals. The most adaptive strategy could even be to commit to new life goals. The results showed that patients with a higher level of optimism also showed higher levels of flexibility in the management of their goals and a higher level of reengagement in new goals. These findings are in line with those of previous research showing that persistence is not a sterile trait in optimistic individuals because they are also more flexible and sensitive to the contextual parameters [31, 35, 56, 57]. In addition, when optimistic individuals repeatedly fail to attain certain goals, they substitute these goals with attainable goals [29, 30, 32] and, in contrast to more pessimistic individuals, they are more likely to report more perceived progress in the pursuit of personal goals [58].

In line with the results of a previous study [29], our results showed that chronic musculoskeletal pain patients who have negative expectations for their future tend to abandon their goals when they think that such goals are unattainable. In contrast to optimism, which was associated with a wider array of strategies to manage goals, pessimism implies a certain rigidity in coping because it is associated only with giving up.

### 4.2. Goal Management Strategies and Affect

Model 1 predicted that affect would mediate optimism/pessimism and goal management strategies. This prediction was not confirmed by the results of this study. Based on the “broaden-and-build theory” [33], it was postulated that positive affect would favour flexible goal management, tenacious goal pursuit, and the reengagement in new goals through the perception of more available alternatives. Negative affect was hypothesized to narrow the perception of available alternatives and consequently to be related to disengagement from unattainable goals.

The findings of this study supported the predictions of Model 2 that positive/negative affect would be the result of goal management strategies. Nevertheless, contrary to the results of a previous longitudinal study using a sample of older adults [36], our results showed that the capacity to withdraw effort and commitment from unattainable goals was not associated with lower negative affect but with higher negative affect. In patients with chronic musculoskeletal pain, the abandonment of cherished life goals may lead to frustration and distress. In contrast, our results and those of previous studies [10–15] have shown that when chronic musculoskeletal pain threatens the patients' life goals but they tenaciously continue to pursue such goals, adapt such goals to the changing circumstances, or commit to new goals, they experience greater emotional well-being.

Finally, our results show that affect did not result from activity patterns, as postulated by Model 3. It seems that positive and negative affect creates the “emotional context” in which activity avoidance and the other activity patterns are performed and negotiated in relation to concomitant life goals [59].

### 4.3. Affect and Activity Patterns

The results of the present study supported Model 2, which hypothesized that affect would be directly related to activity patterns. Specifically, it was found that negative affect was related to the pattern of activity avoidance in which patients give up general activities due to their condition. According to previous studies [6, 7], this activity pattern is associated with the poorest well-being. Positive affect was positively related to task-contingent persistence, which is the activity pattern with the best adaptive results [6, 7]. Task-contingent persistence means that, despite pain, patients carry on with a task or an activity until it is finished. This response suggests that patients show less protective behaviour because the value of other life goals outweighs the value of pain control. Positive affect was also related to the more infrequent use of pain avoidance in which patients interrupt specific actions because of pain.

According to previous research, several processes may explain how positive affect might influence these activity patterns. Firstly, positive affect [60] may help replenish depleted self-regulatory resources, making patients more resistant to activity avoidance. Secondly, positive affect is related to approach goals, which are goals that individuals work toward in order to gain or accomplish something positive, as opposed to goals that seek to avoid a negative outcome [61]. Finally, positive affect may make patients able to “broaden and build” [33], which refers to the act of stepping back to see the larger picture of their lives instead of a pain-centred representation of their lives. This approach would help them to persist in meaningful activities and “avoid avoidance.” These three hypotheses could be the topic of systematic research.

Contrary to the postulates of Models 1 and 3, pain management strategies were not directly related to activity patterns; as mentioned, their relationship was mediated by affect. This may be because the assessment tools used in the present study measure dispositional goal adjustment capacities. There is evidence suggesting that disposition and situational adjustment capacities may operate somewhat differently from each other [62]. Therefore, future research should investigate the relationship between activity patterns and goal adjustment strategies using specific situational measures, such as vignettes, applied to the chronic pain condition. The aforementioned limitation could also account for the fact that only three activity patterns were included in the final model. It may be the case that pain-specific goal management strategies would have shown significant relationships with more activity patterns.

On the other hand, the present approach has undeniable advantages. It emphasizes the role of dispositional variables or, to put it in another way, the role of the history of the patients. The relatively stable expectations for the future and for managing goals that may have existed before the onset of pain appear to be significantly related to activity patterns through affect. If future prospective research replicates these findings, then the assessment of optimism/pessimism and general goal management strategies will enable the early prediction of which patients will develop adaptive or dysfunctional activity patterns.

### 4.4. The Role of Pain Intensity

The results of the present study showed that pain intensity was positively related to negative affect, negatively related to positive affect, and related to activity patterns through these variables. The role of pain intensity in the adaptation of patients cannot be underplayed [63]. Pain and affect are inextricably linked because pain is not only an aversive physical state but also an aversive emotional state in which negative affective responses serve as a protective function motivating the individual to escape imminent threat [64].

### 4.5. Limitations of This Study

The present study has several limitations, and the results should be interpreted accordingly. Firstly, the only method used was self-reporting. Shared method variance may have contributed to the results. Secondly, the cross-sectional nature of the study does not allow causality to be inferred. Thirdly, twenty-one participants were excluded from the sample because they presented anomalous values for one of the variables included in the models. It has been demonstrated that, in most cases, errors of inference are significantly reduced by the removal of outliers [65]; nevertheless, it cannot be discounted with complete certainty that the removal of outliers may negatively affect the representativeness of the sample [66].

### 4.6. Clinical Implications

The findings of the present study demonstrate that, in order to promote adaptive activity patterns in patients with chronic musculoskeletal pain, it is not enough to aim at “fixing what is wrong”: it is also essential to aim at “building what is strong” [67]. In contrast to the conceptualization of positive and negative affect being on the same continuum, research has clearly shown that they are separable affective states [64], which implies that interventions on negative affect do not guarantee improvements in positive affect. That is, patients with deficits in positive affectivity need interventions aimed at augmenting positive affect. One study [64] discussed how cognitive behavioural therapy for pain, acceptance and commitment therapy, and mindfulness-based stress reduction incorporate aspects of positive affect enhancement and encouraged the development of interventions aimed at the generation of positive affect among patients with chronic pain. A similar technique is the Best Possible Self, which aims to increase optimism [68–71]. Finally, a recent study demonstrated the clinical usefulness of an Internet-based positive psychology self-help intervention for the management of chronic pain [72].

The results of the present study suggest that cognitive behavioural intervention programs for individuals with chronic pain may benefit from the inclusion of elements aimed at promoting goal adjustment. The action phase model of goal attainment [73] has been proposed as a useful theoretical framework to integrate motivational strategies in pain intervention programs [74]. Recently, an experimental study showed that implementation intentions reduced escape-avoidance behaviour during painful tasks in healthy individuals [75]. In addition, a short goal-pursuit intervention has been developed to improve physical capacity in patients with chronic pain. This intervention includes problem-solving techniques to overcome obstacles and an implementation intention procedure [76].

### 4.7. Conclusion

The strategies used by patients with chronic musculoskeletal pain to manage their life goals are related to the different ways in which they engage in daily activities. This relationship is mediated by positive/negative affect. Optimism can be regarded as a protective factor that fosters the use of flexible goal management strategies.

## Figures and Tables

**Table 1:** Means, standard deviations (SD), and Pearson correlations.

Variables	Range	Mean (SD)	2^a^	3	4	5	6	7	8	9	10	11	12	13	14	15	16
1. Optimism	0–12	7.68 (2.99)	−0.77^∗∗^	0.67^∗∗^	−0.51^∗∗^	0.46^∗∗^	0.61^∗∗^	−0.51^∗∗^	0.72^∗∗^	−0.27^∗∗^	−0.44^∗∗^	0.18^∗∗^	−0.08	−0.03	0.05	0.14^∗^	−0.02
2. Pessimism	0–12	5.18 (3.12)	—	−0.63^∗∗^	0.48^∗∗^	−0.56^∗∗^	−0.48^∗∗^	0.54^∗∗^	−0.63^∗∗^	0.30^∗∗^	0.55^∗∗^	−0.25^∗∗^	0.11	0.03	−0.03	−0.08	0.09
3. Positive affect	11–45	28.99 (7.73)		—	−0.51^∗∗^	0.53^∗∗^	0.54^∗∗^	−0.58^∗∗^	0.60^∗∗^	−0.38^∗∗^	−0.59^∗∗^	0.36^∗∗^	−0.08	0.03	0.05	0.09	−0.13^∗^
4. Negative affect	10–47	24.27 (7.63)			—	−0.33^∗∗^	−0.40^∗∗^	0.40^∗∗^	−0.45^∗∗^	0.29^∗∗^	0.53^∗∗^	−0.12	0.28^∗∗^	0.16^∗^	−0.02	−0.12	0.04
5. Tenacious goal pursuit	21–60	42.24 (7.95)				—	0.54^∗∗^	−0.57^∗∗^	0.44^∗∗^	−0.27^∗∗^	−0.47^∗∗^	0.33^∗∗^	−0.08	0.11	−0.02	0.06	−0.11
6. Flexible goal adjustment	8–35	23.77 (5.11)					—	−0.32^∗∗^	0.55^∗∗^	−0.17^∗^	−0.29^∗∗^	0.13^∗∗^	−0.03	−0.01	0.01	0.17^∗^	0.01
7. Goal disengagement	5–16	9.60 (2.58)						—	−0.52^∗∗^	0.36^∗∗^	0.51^∗∗^	−0.28^∗∗^	0.11	−0.07	−0.02	0.00	0.08
8. Goal reengagement	5–25	17.14 (3.85)							—	−0.27^∗∗^	−0.44^∗∗^	0.19^∗^	−0.14^∗∗^	−0.06	0.06	0.20^∗∗^	−0.00
9. Pain avoidance	0–12	6.70 (2.64)								—	0.53^∗∗^	−0.70^∗∗^	−0.17^∗^	−0.44^∗∗^	0.41^∗∗^	0.32^∗∗^	0.51^∗∗^
10. Activity avoidance	0–12	6.30 (2.76)									—	−0.48^∗∗^	0.20^∗∗^	−0.04	0.02	−0.05	0.25^∗∗^
11. Task-contingent persistence	0–12	6.08 (2.93)										—	0.17^∗∗^	0.53^∗∗^	−0.43^∗∗^	−0.37^∗∗^	−0.57^∗∗^
12. Pain-contingent persistence	0–12	6.88 (3.26)											—	0.46^∗∗^	−0.11	−0.21^∗∗^	−0.16^∗^
13. Excessive persistence	0–12	5.21 (2.94)												—	−0.46^∗∗^	−0.53^∗∗^	−0.53^∗∗^
14. Pacing to increase activity levels	0–12	6.17 (2.60)													—	0.80^∗∗^	0.73^∗∗^
15. Pacing to conserve energy for valued activities	0–11	6.01 (2.71)														—	0.73^∗∗^
16. Pacing to reduce pain	0–12	6.70 (2.76)															—

^∗^ *P* < 0.05;^∗∗^*P* < 0.01; ^a^the numbers in the upper row correspond to the variables in rows 1 to 12.

**Table 2:** Goodness-of-fit indexes of the three models tested.

	*χ* ^2^/d.f.^a^	NFI	CFI	RMSEA	AIC
Model 1	0.47	0.99	1	0.00	157.06
Model 2	0.39	0.99	1	0.00	144.83
Model 3	0.40	0.99	1	0.00	158.65

*Note*. Model 1: optimism/pessimism → positive/negative affect → goal management strategies → activity patterns; Model 2: optimism/pessimism → goal management strategies → positive/negative affect → activity patterns; Model 3: optimism/pessimism → goal management strategies → activity patterns → positive/negative affect; NFI, Normed Fit Index; CFI, Comparative Fit Index; RMSEA, root mean-square error of approximation; AIC, Akaike Information Criterion; ^a^*χ*^2^/d.f.: Satorra-Bentler chi-square divided by degrees of freedom.

## References

[1] Fisher E., Palermo T. M. (2016). Goal pursuit in youth with chronic pain. *Children*.

[2] Crombez G., Lauwerier E., Goubert L., Van Damme S. (2016). Goal pursuit in individuals with chronic pain: a personal project analysis. *Frontiers in Psychology*.

[3] Stommen N. C., Verbunt J. A., Goossens M. E. (2016). Future goals of adolescents and young adults with chronic musculoskeletal pain. *European Journal of Pain*.

[4] Vlaeyen J. W. S., Linton S. J. (2012). Fear-avoidance model of chronic musculoskeletal pain: 12 years on. *Pain*.

[5] Vlaeyen J. W., Crombez G., Linton S. J. (2016). The fear-avoidance model of pain. *Pain*.

[6] Kindermans H. P., Roelofs J., Goossens M. E., Huijnen I. P., Verbunt J. A., S Vlaeyen J. W. (2011). Activity patterns in chronic pain: underlying dimensions and associations with disability and depressed mood. *Journal of Pain*.

[7] Esteve R., Ramírez-Maestre C., Peters M. L., Serrano-Ibáñez E. R., Ruíz-Párraga G. T., López-Martínez A. E. (2016). Development and initial validation of the activity patterns scale in patients with chronic pain. *Journal of Pain*.

[8] Van Damme S., Kindermans H. P. (2015). A self-regulation perspective on avoidance and persistence behavior in chronic pain: new theories, new challenges?. *Clinical Journal of Pain*.

[9] Brandtstädter J., Renner G. (1990). Tenacious goal pursuit and flexible goal adjustment: explication and age-related analysis of assimilative and accommodative strategies of coping. *Psychology and Aging*.

[10] Kranz D., Bollinger A., Nilges P. (2010). Chronic pain acceptance and affective well-being: a coping perspective. *European Journal of Pain*.

[11] Schmitz U., Saile H., Nilges P. (1996). Coping with chronic pain: flexible goal adjustment as an interactive buffer against pain-related distress. *Pain*.

[12] Van Damme S., de Waegeneer A., Debruyne J. (2016). Do flexible goal adjustment and acceptance help preserve quality of life in patients with multiple sclerosis?. *International Journal of Behavioral Medicine*.

[13] Mens M. G., Wrosch C., Scheier M. F., Whitbourne S. K. (2014). Goal adjustment theory. *The Wiley-Blackwell Encyclopedia of Adult Development and Aging*.

[14] Wrosch C., Scheier M. F., Miller G. E., Schulz R., Carver C. S. (2003). Adaptive self-regulation of unattainable goals: goal disengagement, goal reengagement, and subjective well-being. *Personality and Social Psychology Bulletin*.

[15] Arends R. Y., Bode C., Taal E., van de Laar M. A. (2013). The role of goal management for successful adaptation to arthritis. *Patient Education and Counselling*.

[16] Carver C. S., Scheier M. F., Segerstrom S. C. (2010). Optimism. *Clinical Psychology Review*.

[17] Affleck G., Tennen H., Zautra A., Urrows S., Abeles M., Karoly P. (2001). Women’s pursuit of personal goals in daily life with fibromyalgia: a value-expectancy analysis. *Journal of Consulting and Clinical Psychology*.

[18] Cannella D. T. L., Lobel M., Glass P., Lokshina I., Graham J. E. (2007). Factors associated with depressed mood in chronic pain patients: the role of interpersonal coping resources. *Journal of Pain*.

[19] Ferreira V. M., Sherman A. M. (2007). The relationship of optimism, pain and social support to well-being in older adults with osteoarthritis. *Aging and Mental Health*.

[20] Geers A. L., Wellman J. A., Fowler S. L., Helfer S. G., France C. R. (2010). Dispositional optimism predicts placebo analgesia. *Journal of Pain*.

[21] Goodin B. R., Kronfli T., King C. D., Glover T. L., Sibille K., Fillingim R. B. (2013). Testing the relation between dispositional optimism and conditioned pain modulation: does ethnicity matter?. *Journal of Behavioral Medicine*.

[22] Goodin B. R., Bier S., McGuire L. (2009). Dispositional optimism buffers the negative influence of catastrophizing on pain response. *Journal of Pain*.

[23] Hanssen M. M., Peters M. L., Vlaeyen J. W., Meevissen Y. M., Vancleef L. M. (2013). Optimism lowers pain: evidence of the causal status and underlying mechanisms. *Pain*.

[24] Hanssen M. M., Vancleef L. M., Vlaeyen J. W., Peters M. L. (2014). More optimism, less pain! The influence of generalized and pain-specific expectations on experienced cold-pressor pain. *Journal of Behavioral Medicine*.

[25] Huber A., Suman A. L., Biasi G., Carli C. (2008). Predictors of psychological distress and well-being in women with chronic musculoskeletal pain: two sides of the same coin?. *Journal of Psychosomatic Research*.

[26] Ramírez-Maestre C., Esteve R., López A. E. (2012). The role of optimism and pessimism in chronic pain patients adjustment. *Spanish Journal of Psychology*.

[27] Velasco-Furlong L. V., Zautra A., Peñacoba-Puente C., López-López A., Barjola Valero P. (2010). Cognitive-affective assets and vulnerabilities: two factors influencing adaptation to fibromyalgia. *Psychology and Health*.

[28] Forgeard M. J. C., Seligman M. E. P. (2012). Seeing the glass half full: a review of the causes and consequences of optimism. *Pratiques Psychologiques*.

[29] Rasmussen H. N., Wrosch C., Scheier M. F., Carver C. S. (2006). Self-regulation processes and health: the importance of optimism and goal adjustment. *Journal of Personality*.

[30] Nes L. S., Segerstrom S. C. (2006). Dispositional optimism and coping: a meta-analytic review. *Personality and Social Psychology Review*.

[31] Hanssen M. M., Vancleef L. M., Vlaeyen J. W., Hayes A. F., Schouten E. G. W., Peters M. L. (2015). Optimism, motivational coping and well-being: evidence supporting the importance of flexible goal adjustment. *Journal of Happiness Studies*.

[32] Duke J., Leventhal H., Brownlee S., Leventhal E. A. (2002). Giving up and replacing activities in response to illness. *Journal Gerontology*.

[33] Fredrickson B. L. (2001). The role of positive emotions in positive psychology: the broaden-and-build theory of positive emotions. *American Psychologist*.

[34] Brands I., Stapert S., Köhler S., Wade D., van Heugten C. (2014). Life goal attainment in the adaptation process after acquired brain injury: the influence of self-efficacy and of flexibility and tenacity in goal pursuit. *Clinical Rehabilitation*.

[35] Segerstrom S. C., Nes L. S. (2006). When goals conflict but people prosper: the case of dispositional optimism. *Journal of Research in Personality*.

[36] Dunne E., Wrosch C., Miller G. E. (2011). Goal disengagement, functional disability, and depressive symptoms in old age. *Health Psychology*.

[37] Esteve R., López-Martínez A. E., Peters M. L. (2017). Activity pattern profiles: relationship with affect, daily functioning, impairment and variables related with life goals. *Journal of Pain*.

[38] Otero J. M., Luengo A., Romero E., Gómez J. A., Castro C. (1988). *Psicología de la Personalidad. Manual de Prácticas*.

[39] Scheier M. F., Carver C. S., Bridges M. W. (1994). Distinguishing optimism from neuroticism (and trait anxiety, self-mastery, and self-esteem): a reevaluation of the life orientation test. *Journal of Personality and Social Psychology*.

[40] Ferrando P. J., Chico-Librán E., Tous J. M. (2002). Propiedades psicométricas del test de optimismo life orientation test. *Psicothema*.

[41] Sandín B., Chorot P., Lostao L., Joiner T. E., Santed M. A., Valiente R. M. (1999). Escalas PANAS de afecto positivo y negativo: validación factorial y convergencia transcultural. *Psicothema*.

[42] Sandín B., Valiente R. M., Chorot P., Sandín B. (2008). Instrumentos para la evaluación del estrés psicosocial. *El Estrés Psicosocial: Conceptos y Consecuencias Clínicas*.

[43] Watson D., Clark L. A., Tellegen A. (1988). Development and validation of brief measures of positive and negative affect: the PANAS scales. *Journal of Personality and Social Psychology*.

[44] Jensen M. P., Turner P., Romano J. M., Fischer L. D. (1999). Comparative reliability and validity of chronic pain intensity measures. *Pain*.

[45] Soubier E., Esteve R., Ramírez-Maestre C. (2017). Adaptación de las escalas tenacious goal pursuit and flexible goal adjustment y goal disengagement and goal reengagement. *Escritos de Psicología*.

[46] Jöreskog K. G., Sörbom D. (1993). *Lisrel 8: Structural Equation Modeling with the SIMPLIS Command Language*.

[47] Campello R. J. G. B., Moulavi D., Zimek A., Sander J. (2015). Hierarchical density estimates for data clustering, visualization, and outlier detection. *ACM Transactions on Knowledge Discovery from Data*.

[48] Batista J. M., Coenders G. (2000). *Modelos de Ecuaciones Estructurales*.

[49] Bentler P. M. (1990). Comparative fit indexes in structural models. *Psychological Bulletin*.

[50] Bentler P. M., Bonnet D. G. (1980). Significance tests and goodness of fit in the analysis of covariance structures. *Psychological Bulletin*.

[51] Akaike H. (1987). Factor analysis and AIC. *Psychometrika*.

[52] Kline R. B. (2005). *Principles and Practice of Structural Equation Modelling*.

[53] Hu L., Bentler P. M. (1999). Cutoff criteria for fit indexes in covariance structure analysis: conventional criteria versus new alternatives. *Structural Equation Modelling*.

[54] Hu L., Bentler P. M. (1998). Fit indices in covariance structure modelling: sensitivity to underparameterized model misspecification. *Psychological Methods*.

[55] Gibson B., Sanbonmatsu D. M. (2004). Optimism, pessimism, and gambling: the downside of optimism. *Personality and Social Psychology Bulletin*.

[56] Monzani D., Steca P., Greco A., D’Addario M., Pancani L., Cappelletti E. (2015). Effective pursuit of personal goals: the fostering effect of dispositional optimism on goal commitment and goal progress. *Personality and Individual Differences*.

[57] Geers A. L., Wellman J. A., Lassiter G. D. (2009). Dispositional optimism and engagement: the moderating influence of goal prioritization. *Journal of Personality and Social Psychology*.

[58] Pavlova M. K., Silbereisen R. K. (2013). Dispositional optimism fosters opportunity-congruent coping with occupational uncertainty. *Journal of Personality*.

[59] Karsdorp P. A., Vlaeyen J. W. (2011). Goals matter: both achievement and pain-avoidance goals are associated with pain severity and disability in patients with low back and upper extremity pain. *Pain*.

[60] Tice D. M., Baumeister R. F., Shmueli D., Muraven M. (2007). Restoring the self: positive affect helps improve self-regulation following ego depletion. *Journal of Experimental Social Psychology*.

[61] Elliot A. J., Thrash T. M. (2002). Approach-avoidance motivation in personality: approach and avoidance temperaments and goals. *Journal of Personality and Social Psychology*.

[62] Mens M. G., Scheier M. (2015). The benefits of goal adjustment capacities for well-being among women with breast cancer: potential mechanisms of action. *Journal of Personality*.

[63] Crombez G., Eccleston C., Van Damme S., Vlaeyen J. W., Karoly P. (2012). Fear-avoidance model of chronic pain: the next generation. *Clinical Journal of Pain*.

[64] Finan P. H., Garland E. L. (2015). The role of positive affect in pain and its treatment. *Clinical Journal of Pain*.

[65] Osborne J. W., Overbay A. (2004). The power of outliers (and why researchers should ALWAYS check for them). *Practical Assessment, Research and Evaluation*.

[66] Orr J. M., Sackett P. R., DuBois C. L. Z. (1991). Outlier detection and treatment in I/O psychology: a survey of researcher beliefs and an empirical illustration. *Personnel Psychology*.

[67] Boselie J. J., Vlaeyen J. W. (2017). Broadening the fear-avoidance model of chronic pain?. *Scandinavian Journal of Pain*.

[68] Meevissen Y. M., Peters M. L., Alberts H. J. (2011). Become more optimistic by imagining a best possible self: effects of a two week intervention. *Journal of Behavior Therapy and Experimental Psychiatry*.

[69] Meevissen Y., Peters M. L., Alberts H. J. (2012). Overcoming ego depletion: the effects of an optimism manipulation on repeated acts of self-control. *BMC Complementary and Alternative Medicine*.

[70] Peters M. L., Flink I. K., Boersma K., Linton S. J. (2010). Manipulating optimism: can imagining a best possible self be used to increase positive future expectancies?. *Journal of Positive Psychology*.

[71] Peters M. L., Meevissen Y. M., Hanssen M. M. (2013). Specificity of the best possible self intervention for increasing optimism: comparison with a gratitude intervention. *Terapia Psicológica*.

[72] Peters M. L., Smeets E., Feijge M. (2017). Happy despite pain: a randomized controlled trial of an 8-week Internet-delivered positive psychology intervention for enhancing well-being in patients with chronic pain. *Clinical Journal of Pain*.

[73] Gollwitzer P. M., Sheeran P. (2006). Implementation intentions and goal achievement: a meta-analysis of effects and processes. *Advances in Experimental and Social Psychology*.

[74] Schrooten M. G., Vlaeyen J. W., Morley S. (2012). Psychological interventions for chronic pain: reviewed within the context of goal pursuit. *Pain Management*.

[75] Karsdorp P. A., Geenen R., Kroese F. M., Vlaeyen J. W. (2016). Turning pain into cues for goal-directed behavior: implementation intentions reduce escape-avoidance behavior on a painful task. *Journal of Pain*.

[76] Christiansen S., Oettingen G., Dahme B., Klinger R. (2010). A short goal-pursuit intervention to improve physical capacity: a randomized clinical trial in chronic back pain patients. *Pain*.

